# Association of A1538G and C2437T single nucleotide polymorphisms in heat shock protein-70 genes with diabetic nephropathy among South Indian population

**DOI:** 10.1042/BSR20160605

**Published:** 2017-03-27

**Authors:** Umapathy Dhamodharan, Krishnamoorthy Ezhilarasi, Balashanmugam Ponjayanthi, Dornadula Sireesh, Kunka Mohanram Ramkumar, Vijay Viswanathan

**Affiliations:** 1 Life Science Division, SRM Research Institute, SRM University, Kattankulathur, Chennai-603203, India; 2 Department of Biochemistry and Molecular Genetics, Prof. M. Viswanathan Diabetes Research Centre and M.V. Hospital for Diabetes (A WHO Collaborating Centre for Research, Education & Training in Diabetes), Royapuram, Chennai-600013, India

**Keywords:** Diabetic Nephropathy, Heat-shock proteins, Microalbuminuria, Macroalbuminuria, Single Nucleotide Polymorphism, Type 2 Diabetes Mellitus

## Abstract

Diabetic Nephropathy (DN) is the leading cause of end-stage renal disease, characterized by progressive albuminuria and conferring additional risk of cardiovascular disease (CVD) and mortality. The crucial role of heat-shock proteins (HSPs) on renal function in patients with DN has been well documented. The present study was aimed to understand the association of *HSP-70* gene variants on the susceptibility of Type 2 Diabetes Mellitus (T2DM) and DN. A total of 946 subjects (549 Males; 397 Females) were recruited and divided into four groups according to the levels of urinary albumin excretion (UAE): those with normoalbuminuria (UAE <30 mg/24 h; *n*=230), those with microalbuminuria (30≤ UAE ≤300 mg/24 h; *n*=230), and those with macroalbuminuria (UAE> 300 mg/24 h; *n*=230). The control group randomly enrolled a consecutive population of 256 healthy subjects who had a routine medical check-up in our hospital. Those subjects had no history or clinical symptoms of diabetes. Subjects were genotyped for *HSP70-2* (+1538 A/G; rs2763979) and *HSP70-hom* (+2437 C/T; rs2227956) by PCR-restriction fragment length polymorphism (RFLP). The ‘G’ allele of *HSP70-2* (+1538 A/G) single nucleotide polymorphism (SNP) showed relative risk for normoalbuminuria, microalbuminuria and macroalbuminuria subjects whereas the ‘T’ allele of *HSP70-hom* (+2437 C/T) SNP showed significant protection against macroalbuminuria subjects. In conclusion, our results indicate that the *HSP70-2* (+1538 A/G) and *HSP70-hom* (+2437 C/T) SNPs are highly associated with renal complications in T2DM among the South Indian population.

## Introduction

Diabetic Nephropathy (DN) is the leading cause of diabetes-related morbidity and mortality and its prevalence is rising worldwide over the past decades. Approximately 40% of the Type 2 Diabetes Mellitus (T2DM) population develops DN and the major manifestations are albuminuria and/or decreased glomerular filtration rate [1]. DN is categorized into three different stages such as normoalbuminuria, microalbuminuria and macroalbuminuria. The prevalence of microalbuminuria, which is considered as a predictor of DN has been reported to be 26.9% in South Indians [2]. Several cross-sectional and prospective studies have been demonstrated that hyperglycaemia, dyslipidaemia, race, genetic and environmental factors are the important contributors for DN [3]. Numerous single nucleotide polymorphisms (SNPs) in the candidate genes have been documented in relation to diabetic complications [4–6 ]. Oxidative stress plays a pivotal role in the development and progression of DN [7]. It has been demonstrated that excess reactive oxygen species (ROS) cause apoptosis of podocytes and its depletion at the onset of DN [8]. Various mechanisms are involved in the adaptive cellular responses which include functional chaperons, antioxidant production and regulation of cellular signals for protein synthesis to survive under oxidative stress conditions [9]. Numerous oxidative stress-related genes are positional candidates, determined by genome-wide association studies (GWAS) and candidate genes studies have confirmed the association of their SNPs with DN [10].

Heat-shock proteins (HSPs) act as molecular chaperones playing an important role in normal cell processes and they help in protein folding, assembly, disassembly and translocation of other proteins. These HSPs are classified by their molecular weight to which 70 kDa HSP-70 represents a wide family, and are associated with cell protection by inhibiting apoptosis through the suppression of c-jun N-terminal kinase (JNK) [11]. Thus, intracellular HSP-70 delays the progression of chronic kidney disease through its cytoprotective action [12]. HSPs have also been reported to be involved in diabetes through their effect on insulin sensitivity [13], and also there are few literatures that relate abnormalities of HSPs and T2DM [14,15]. Also, there are a few earlier studies that have documented the crucial role of HSPs in renal cell survival and matrix remodelling in several acute and chronic renal diseases [16].

In humans, the HSP-70 family is encoded by *HSP70-1* (*HSPA1A*), *HSP70-2* (*HSPA1B*) and *HSP70-hom* (*HSPA1L*) mapped within the MHC class III region (6p21.3). *HSP70-1* and *HSP70-2* encode an identical heat-inducible protein HSP-70 and they differ in their regulatory domains, whereas *HSP70-hom* encodes a non-heat-inducible form [17]. *HSP-70* gene SNPs have been found to be risk factors in several human disorders including Parkinson’s disease [18], spondyloarthropathies [19], sarcoidosis [20], ulcerative colitis, Crohn’s disease [21 ] and other diseases [22]. SNPs were selected based on the HapMap database (http://hapmap.ncbi.nlm.nih.gov/) and dbSNP (http://www.ncbi.nlm.nih.gov/snp/). Inclusion criteria were as follows: SNPs located in the entire region of the *HSP70* genes (*HSPA1A*, *HSPA1B* and *HSPA1L*) with a minor allele frequency (MAF) >0.10 in South Asian population, covering 2-kb upstream of the transcription start site and all exons and introns. As a pilot study, we selected *HSP70-2* (*HSPA1B*) that is located in the 5′-flanking region and *HSP70-hom* (*HSPA1L*) that lies on exon-2, however for the present pilot study, we ruled out *HSPA1A* because it was in the 5′-UTR region.

Therefore, in the present study, we made an attempt to investigate the association of *HSP70-2* (+1538 A/G) rs2763979 and *HSP70-hom* (+2437 C/T) rs2227956 SNPs on the susceptibility of DN among the South Indian population.

## Materials and methods

### Study population

A total of 946 subjects, 256 normal glucose tolerant (non-diabetic controls, NGT) subjects as Group-I, 230 T2DM subjects without nephropathy (normoalbuminuria, KDM) as Group-II, 230 T2DM subjects with microalbuminuria as Group-III and 230 T2DM subjects with macroalbuminuria as Group-IV were included in the present study. NGT subjects were randomly selected healthy volunteers (mostly blood donors and hospital staff) with no history of diabetes and renal or cardiovascular diseases (CVDs). Subjects with fasting plasma glucose (FPG) <5.6 mmol/l (100 mg/dl) and 2 h postprandial plasma glucose (PPG) value ≤7.8 mmol/l (140 mg/dl) during an oral glucose tolerance test [23] formed the NGT group. The patients were selected from the outpatient department of M.V. Hospital for Diabetes, Chennai, a tertiary care centre in India. T2DM was defined as the FPG level of ≥7.0 mmol/l (126 mg/dl) and/or PPG level of ≥11.1 mmol/l (200 mg/dl) [23]; T2DM subjects with duration of diabetes >10 years and negative for urinary protein (proteinuria) and having urinary albumin levels (<30 μg/mg of creatinine) measured on two consecutive occasions formed the KDM group. Subjects with urinary albumin excretion (UAE) 30–300 and ≥300 μg/mg of creatinine (measured by immunoturbidometric assay) in at least two out of three fasting urine collections over a period of 3 months formed the microalbuminuria and macroalbuminuria groups respectively, along with the presence of diabetic retinopathy in the latter group. All the subjects included in the present study were unrelated individuals of South Indian origin. The study was approved by the institutional ethical committee and written informed consent was obtained from all subjects enrolled in the study in accordance with principles of the Declaration of Helsinki. History of diabetic ketoacidosis or hypoglycaemic coma in the past 3 months preceding the study, presence of urinary tract infection, other renal disease, rheumatological, neoplastic, other endocrine diseases (except diabetes) was the exclusion criteria. Subjects on antihypertensives, statins or using immunomodulatory medications were also excluded from the study. Anthropometric and demographic details like age, weight, height, duration of diabetes were recorded for all the study subjects. Biochemical analyses were carried out on a Hitachi-912 Autoanalyzer (Hitachi, Mannheim, Germany) using commercial kits (Roche Diagnostics, Mannheim, Germany). Estimation of FPG, serum cholesterol, serum triglycerides (TGL), HDL cholesterol (HDL-c) and LDL cholesterol (LDL-c) were standard methods as described earlier [24].

### Sample size calculation and power of study

A pilot study was conducted using 50 subjects per group. Based on the findings of the preliminary results, with a 95% confidence interval (CI), an estimated *P* value of <0.05 and a power of 80%, the present sample size was derived.

### Genotyping and functional prediction tools

Genomic DNA was extracted from peripheral blood using the standard protocol as described by Maniatis et al. [25] Genotypes for *HSP70-2* (+1538 A/G) and *HSP70-hom* (+2437 C/T) SNPs were determined by PCR followed by restriction fragment length polymorphism (RFLP). The primer sequences, amplicon sizes and the restriction enzymes used are given in Table 1.

**Table 1:** Details of primer sequences, amplicon sizes and the restriction enzymes

S. No.	Gene symbol	SNP position	Reference SNP number	Primer sequences	PCR product size (bp)	Restriction enzymes	Expected band patterns (bps)
1.	*HSP70-2*	+1538 A/G	*rs2763979*	F: 5′- ACCCTGGAGCCCGTGGAGAA-3′	383	PstI	383, 244 and 139
				R: 5′- CACCCGCCCGCCCCGTAGG-3′			
2.	*HSP70-hom*	+2437 C/T	*rs2227956*	F: 5′- GGACAAGTCTGAGAAGGTACAG-3′	705	NcoI	705, 550 and 155
				R: 5′- GTAACTTAGATTCAGGTCTGG-3′			

F, forward; R, reverse.

Numerous computational techniques have been recently developed to predict the functional significance of amino acid substitutions; here, we use the PolyPhen-2 programme (http://genetics.bwh.harvard.edu/pph2/) and SIFT (http://sift.bii.a-star.edu.sg/) to categorize the mutation. The PolyPhen-2 output was divided into five categories: probably benign (0.000–0.999), borderline (1.000–1.249), potentially damaging (1.250–1.449), possibly damaging (1.500–1.999) and probably damaging (≥2.000) [26].

### Statistical analysis

Statistical calculations were performed using SPSS (version 20.0; SPSS, Chicago, IL, U.S.A.). Normally distributed data are presented as mean ± S.D. The Hardy–Weinberg equilibrium was tested with the χ^2^ test. Genotype distribution and allele frequencies were compared among groups using a χ^2^ test of independence with 2 × 2 contingency and z statistics. A Student’s *t* test and a Mann–Whitney U test were used to determine the statistical significance. Where appropriate, the odds ratio (OR) with 95% CI were calculated. A two-tailed type I error rate of 5% was considered statistically significant.

## Results

### Clinical and biochemical characteristics of the study subjects

Selected clinical and biochemical characteristics of study subjects are presented in Table 2. Parameters such as Age, Systolic Blood Pressure (SBP), FPG, PPG, HbA1c, urea and creatinine were found to be significantly higher whereas body mass index (BMI), HDL-c and LDL-c were significantly lowered in albuminuric subjects (micro + macro), when compared with normoalbuminuria subjects. Age, FPG and PPG were significantly elevated in microalbuminuria subjects when compared with subjects having normoalbuminuria. Similarly, when macroalbuminuria subjects were analysed separately, the levels of FPG, PPG, HbA1c, total serum cholesterol, TGL, urea and creatinine were found to be significantly higher than the normoalbuminuria subjects. The number of subjects with other complications of diabetes such as DN, Peripheral Vascular Disease (PVD) and Coronary Artery Disease (CAD) were significantly higher in the macroalbuminuria (56.4%, 10.2% and 15.8% respectively) as compared with the normoalbuminuria subjects (40.4%, 6.9% and 11.5%) respectively. With respect to treatment regimen, nearly 57.1% of macroalbuminuria subjects were on combination of oral hypoglycaemic agents (OHA) and insulin therapy, whereas 54.5% of normoalbuminuria and 50.0% of microalbuminuria subjects were on OHA. All cases and control subjects were genotyped for the *HSP70-2* (+1538 A/G) and *HSP70-hom* (+2437 C/T) SNPs.

**Table 2:** Clinical and biochemical characteristics of the study subjects

Clinical parameters (*n*=946)	NGT (*n*=256)	Normoalbuminuria (*n*=230)	Albuminuric (micro + macro)^†^ (*n*=460)	Microalbuminuria^‡^ (*n*=230)	Macroalbuminuria^§^ (*n*=230)
Gender (M/F)	**134/122**	**128/102**	**287/173**	**138/92**	**149/81**
Age (years)	41.9 ± 8.7	49.3 ± 9.5***	53.8 ± 10.6***	52.3 ± 10.6*	55.4 ± 10.5***
BMI (kg/m^2^)	25.6 ± 4.2	27.3 ± 4.4***	26.3 ± 4.3**	26.5 ± 4.6	26.1 ± 4.0**
SBP (mmHg)	116.0 ± 13.0	126.5 ± 17.2***	129.1 ± 16.8*	128.4 ± 15.5	129.7 ± 18.0*
Diastolic BP (mmHg)	77.4 ± 7.4	81.5 ± 9.2***	80.1 ± 8.7	80.2 ± 7.8	80.1 ± 9.6
FPG (mg/dl)	97.2 ± 8.8	173.9 ± 72.3***	186.1 ± 71.5**	199.1 ± 78.4***	207.4 ± 101.8***
PPG (mg/dl)	110.5 ± 18.0	272.6 ± 96.4***	320.7 ± 108.5***	314.6 ± 108.1***	328.4 ± 117.4***
Glycated haemoglobin (%)	5.3 ± 0.2	8.8 ± 5.5***	9.6 ± 2.4***	9.3 ± 2.3	9.9 ± 2.5**
Total serum cholesterol (mg/dL)	182.5 ± 36.8	187.8 ± 44.0	189.0 ± 48.7***	189.7 ± 47.7	192.3 ± 38.9**
Serum TGLs (mg/dl)	117.0 ± 47.3	156.1 ± 69.7***	154.5 ± 71.7	157.8 ± 81.8	171.4 ± 99.7***
HDL-c (mg/dl)	44.1 ± 9.0	44.7 ± 9.4	38.8 ± 11.2***	42.6 ± 9.5	34.08 ± 11.3***
LDL-c (mg/dl)	109.0 ± 29.7	115.9 ± 36.8	101.7 ± 34.7***	112.5 ± 36.3	110.1 ± 34.9
VLDL-cholesterol (mg/dl)	29.8 ± 15.0	27.2 ± 13.9	27.5 ± 13.6	26.4 ± 13.5	28.7 ± 13.7
Urea (mg/dl)	20.5 ± 6.0	22.7 ± 5.6	32.2 ± 15.9***	25.5 ± 9.0	39.3 ± 18.4***
Creatinine (mg/dl)	0.8 ± 0.1	0.9 ± 0.1	1.1 ± 0.5***	0.9 ± 0.2	1.3 ± 0.6***

All data are reported as mean ± S.D. VLDL, very low density lipoprotein.

*P<0.05; **P<0.01; ***P<0.001.

^†^indicates comparison was made between normoalbuminuria and albuminuric (microalbuminuria + macroalbuminuria).

^‡^indicates comparison was made between normoalbuminuria and microalbuminuria.

^§^indicates comparison was made between normoalbuminuria and macroalbuminuria.

### Genetic association of *HSP70-2* (+1538 A/G) SNP with disease phenotype

The genotype and allele frequencies of *HSP70-2* (+1538 A/G) and *HSP70-hom* (+2437 C/T) SNPs in the study subjects have been shown in Table 3. The genotypic distribution of these SNPs was found to be in Hardy-Weinberg equilibrium. The minor ‘G’ allele frequency of *HSP70-2* (+1538 A/G) SNP were found to be significantly higher in albuminuric subjects (56.2%), followed by normoalbuminuria (54.8%) when compared with NGT subjects (*P*<0.01) (Table 3). The ‘G’ allele frequency was found as 56.7% and 55.7% in microalbuminuria and macroalbuminuria subjects respectively. Logistic regression analysis showed that GG genotype conferred significant risk for macroalbuminuria (OR: 8.4; 95% CI: 2.2–32.6; *P*<0.01), for microalbuminuria (OR: 5.4; 95% CI: 1.8–16.6; *P*<0.01) and for normoalbuminuria (OR: 5.2; 95% CI: 1.6–16.5; *P*<0.05) over the AA genotype as determined by various models after adjusting for the potential confounders for DN such as age, gender and BMI. The unadjusted OR for ‘G’ allele in microalbuminuria subjects was found as 1.7 (95% CI: 1.2–2.3; *P*<0.001), for macroalbuminuria it was 1.6 (95% CI: 1.2–2.3; *P*<0.01) and 1.5 (95% CI: 1.1–2.0; *P*<0.05) for normoalbuminuria, when compared with the ‘A’ allele of NGT subjects (Table 4).

**Table 3:** Distribution of genotype and allele frequencies of *HSP70-2* and *HSP70-hom* SNPs in the study subjects

SNP (*n*=946)	Genotype/allele	NGT (*n*=256)	Normoalbuminuria (*n*=230)	Albuminuric (micro/macro) (*n*=460)	Microalbuminuria (*n*=230)	Macroalbuminuria (*n*=230)
***HSP70-2* (+1538 A/G)**	**AA**	49 (19.1%)	32 (13.9%)	75 (16.3%)	38 (16.5%)	37 (16.1%)
	**AG**	170 (66.4%)	144 (62.6%)	253 (55.0%)	123 (53.5%)	130 (56.5%)
	**GG**	37 (14.5%)	54 (23.5%)	132 (28.7%)	69 (30.0%)	63 (27.4%)
	**A**	268 (52.3%)	208 (45.2%)	403 (43.8%)	199 (43.3%)	204 (44.3%)
	**G**	244 (47.7%)	252 (54.8%)	517 (56.2%)	261 (56.7%)	256 (55.7%)
***HSP70-hom* (+2437 C/T)**	**CC**	22 (8.6%)	34 (14.8%)	77 (16.7%)	36 (15.7%)	41 (17.8%)
	**CT**	60 (23.4%)	62 (27.0%)	170 (37.0%)	80 (34.8%)	90 (39.2%)
	**TT**	174 (68.0%)	134 (58.2%)	213 (46.3%)	114 (49.5%)	99 (43.0%)
	**C**	104 (20.3%)	130 (28.3%)	324 (35.2%)	152 (33.0%)	172 (37.4%)
	**T**	408 (79.7%)	330 (71.7%)	596 (64.8%)	308 (67.0%)	288 (62.6%)

Values are numbers (percentage). NGT, subjects with normal glucose tolerance; normoalbuminuria.

**Table 4:** Association of *HSP70-2* and *HSP70-hom* gene SNPs with DN: ORs for minor alleles and their homo- and heterozygous genotypes

SNP (*n*=946)	NGT compared with Normoalbuminuria		NGT compared with Microalbuminuria		NGT compared with Macroalbuminuria	
	Unadjusted OR (95% CI)	Adjusted OR (95% CI)^†^	Unadjusted OR (95% CI)	Adjusted OR (95% CI)^†^	Unadjusted OR (95% CI)	Adjusted OR (95% CI)^†^
***HSP70-2* (+1538 A/G)**						
**AA**	Ref.		Ref.		Ref.	
**AG**	4.2** (1.7–10.4)	2.8* (1.0–7.4)	2.3** (1.0–4.9)	1.8 (0.8–4.6)	4.3** (1.6–11.3)	2.9 (0.9–9.7)
**GG**	7.3*** (2.6–20.7)	5.2* (1.6–16.5)	6.9*** (2.8–17.3)	5.4** (1.8–16.6)	9.7*** (3.3–29.0)	8.4** (2.2–32.6)
**Minor ‘G’ allele**	1.5* (1.1–2.0)	–	1.7*** (1.3–2.4)	–	1.6** (1.2–2.3)	–
***HSP70-hom* (+2437 C/T)**						
**CC**	Ref.		Ref.		Ref.	
**CT**	0.6 (0.2–1.9)	0.6 (0.1–2.2)	0.9 (0.3–3.7)	0.7 (0.2–2.9)	0.9 (0.3–2.6)	0.8 (0.1–3.9)
**TT**	0.5 (0.2–1.5)	0.4 (0.1–1.6)	0.5 (0.1–1.44	0.3 (0.1–1.1)	0.3* (0.1–0.8)	0.2* (0.1–0.9)
**Minor ‘T’ allele**	0.7 (0.5–1.1)	–	0.5 (0.4–0.8)	–	0.4*** (0.3–0.6)	–

Figures in bold were significant (*P*<0.05).

*P<0.05; **P<0.01; ***P<0.001.

^†^OR adjusted for confounding factor (age, gender and BMI).

The OR was computed taking the normoalbuminuria subjects as reference instead of NGT (Table 4a). Our results showed that the GG genotype conferred significant risk for albuminuric subjects (OR: 1.4; 95% CI: 0.9–2.4; *P*<0.01). The unadjusted OR for ‘G’ allele in albuminuric subjects (micro + macro) subjects was found as 1.1 (95% CI: 0.8–1.3; *P*<0.01), when compared with ‘A’ allele of normoalbuminuria subjects. Further, logistic regression was analysed between the individual subgroups (i.e. microalbuminuria and macroabluminuria) and results showed that the ‘G’ allele conferred significant risk for macroabluminuria subjects (OR: 1.1; 95% CI: 0.8–1.5; *P*<0.01), when compared with ‘A’ allele of normoalbuminuria subjects. However, no significant association was observed with respect to risk for microalbuminuria. Overall the ‘G’ allele of *HSP70-2* (+1538 A/G) SNP was associated with normoalbuminuria and showed a significant risk for macroalbuminuria subjects. Moreover, the clinical and biochemical characteristics of the normoalbuminuria, microalbuminuria and macroalbuminuria subjects in relation to AA, AG and GG genotypes of *HSP70-2* (+1538 A/G) were compared and found that the GG genotype in normoalbuminuria subjects had higher levels of FPG, HbA1c and LDL-c, when compared with AA genotype. In the microalbuminuria subjects, the GG genotype showed significantly higher levels of FPG, PPG and TGL. In the macroalbuminuria subjects, the GG genotype had higher levels of serum TGL and significantly lower levels of HDL-c (Supplementary Table 1a). This result suggests that the observed effect of the (+1538 A/G) SNP of *HSP70-2* might be associated with cardiovascular risk factors.

**Table 4a:** Association of *HSP70-2* and *HSP70-hom* gene SNPs in relation to albuminuria using logistic regression analysis

SNP *(n*=946)	Normoalbuminuria compared with Microalbuminuria		Normoalbuminuria compared with Macroalbuminuria		Normoalbuminuria compared with Albuminuria (micro + macro)	
	Unadjusted OR (95% CI)	Adjusted OR (95% CI)^†^	Unadjusted OR (95% CI)	Adjusted OR (95% CI)^†^	Unadjusted OR (95% CI)	Adjusted OR (95% CI)^†^
***HSP70-2 (+1538 A/G)***						
**AA**	Ref.		Ref.		Ref.	
**AG**	0.5 (0.2–1.6)	0.5 (0.2–1.5)	1.0 (0.3–3.4)	0.8 (0.2–3.0)	0.7 (0.3–1.8)	0.6 (0.2–1.7)
**GG**	0.9 (0.3–2.9)	0.9 (0.3–3.0)	1.3** (0.4–4.8)	1.1** (0.3–4.1)	1.5** (0.9–2.5)	1.4** (0.9–2.4)
**Minor ‘G’ allele**	1.1 (0.8–1.6)	–	1.1** (0.8–1.5)	–	1.1** (0.8–1.3)	–
***HSP70-hom (+2437 C/T)***						
**CC**	Ref.		Ref.		Ref.	
**CT**	1.6 (0.5–4.9)	1.5 (0.5–4.7)	1.4 (0.5–4.0)	1.3 (0.4–3.9)	1.5 (0.6–3.8)	1.4 (0.5–3.6)
**TT**	0.9 (0.3–2.6)	0.7 (0.2–2.1)	0.6 (0.2–1.5)	0.5 (0.2–1.4)	0.5** (0.3–0.7)	0.4** (0.2–0.7)
**Minor ‘T’ allele**	0.7 (0.5–1.1)	–	0.5** (0.3–0.8)	–	0.7** (0.6–0.9)	–

Figures in bold were significant (*P*<0.05).

*P<0.05; **P<0.01.

^†^OR adjusted for confounding factor (age, gender and BMI).

### Genetic association of *HSP70-hom* (+2437 C/T) SNP with disease phenotype

For *HSP70-hom* (+2437 C/T) SNP, the frequency of minor ‘T’ allele were found to be significantly decreased in albuminuric subjects (64.8%), followed by normoalbuminuria (71.7%), when compared with NGT subjects (*P*<0.01) (Table 3). The ‘T’ allele frequency was 67.0% and 62.6% in microalbuminuria and macroalbuminuria subjects respectively. Logistic regression analysis showed that TT genotype conferred significant protection against macroalbuminuria subjects (OR: 0.2; 95% CI: 0.1–0.9; *P*<0.05), when compared with CC (the reference genotype) of NGT subjects. The unadjusted OR for ‘T’ allele in macroalbuminuria subjects was (OR: 0.4; 95% CI: 0.3–0.6; *P*<0.001), when compared with ‘C’ allele of NGT subjects (Table 4). When the OR was computed taking the normoalbuminuria subjects as reference instead of NGT, the ‘T’ allele again showed significant protection for albuminuric subjects (OR: 0.7; 95% CI: 0.6–0.9; *P*<0.01) (Table 4a). Further, logistic regression was analysed between the individual subgroups (i.e. microalbuminuria and macroabluminuria). Our results showed that ‘T’ allele conferred significant protection against macroabluminuria subjects (OR: 0.5; 95% CI: 0.3–0.8; *P*<0.01), when compared with ‘A’ allele of normoalbuminuria subjects. However no significant association was observed with respect to risk for microalbuminuria. Overall the ‘T’ allele of *HSP70-hom* (+2437 C/T) SNP showed protection against macroalbuminuria subjects. The clinical and biochemical characteristics compared within the three genotypes of *HSP70-hom* showed that the TT genotype had significantly lower BMI, serum cholesterol, TGL and LDL-c in normoalbuminuria subjects. In the microalbuminuria subjects, the TT genotype showed significantly lower HbA1c and higher HDL-c, when compared with CC genotype and macroalbuminuria subjects showed significantly lower urea levels in the TT genotype (Supplementary Table 1b).

Results of the functional prediction analysis for the non-synonymous variant (rs2227956 of *HSP-70 hom*) showed PolyPhen score of 0.052 with a sensitivity of 0.93 and specificity of 0.63 and the mutation was predicted to be benign/tolerated, whereas the SIFT analysis also predicted this mutation to be tolerated with a SIFT score of 0.098, whereas the other variant studied (rs2763979 of *HSP70-2*) is located in the 5′-flanking region (Supplementary Table 2).

## Discussion

DN is a chronic disorder typically characterized by progressive albuminuria and a decline in renal function. Few linkage studies and GWAS evidenced for a genetic susceptibility to DN [27]. A genetic component of diabetes and its complications (including DN) is obvious, but the causative genes and mechanisms have not yet been satisfactorily identified. In the present study, we studied the genetic variations in *HSP-70* gene for their association towards different stages of DN subjects. The current study revealed that the ‘G’ allele of *HSP70-2* (+1538 A/G) SNP is associated with a significant risk for normoalbuminuria subjects. With respect to DN, the ‘G’ allele of this SNP conferred risk only for macroalbuminuria subjects, whereas the ‘T’ allele of *HSP70-hom* (+2437 C/T) SNP conferred significant protection against macroalbuminuria subjects, but not with normoalbuminuria or microalbuminuria subjects.

The SNPs in the *HSP-70* gene have been reported in different populations and were positively correlated with the level of circulatory cytokines in response to inflammatory stimuli [21,28]. SNPs within *HSP70-2* and *HSP70-hom* have been first characterized by Milner and Campbell [17]. Few studies highlighting the important role of HSP-70 in T1DM with DN [29], T2DM [30] and cardiovascular events in T2DM patients [31]. In the present study, all 946 subjects were genotyped for *HSP70-2* and *HSP70-hom* SNPs in different stages of DN subjects. In the *HSP70-2* (+1538 A/G) SNP, we observed that the ‘G’ allele under both homo (GG) and heterozygotic (AG) conditions conferred significant risk for normoalbuminuria subjects, whereas with respect to DN the GG genotype and the ‘G’ allele of *HSP70-2* (+1538 A/G) SNP conferred risk for macroalbuminuria subjects. Thus, the results of present study showed that SNP in ‘G’ allele of *HSP70-2* (+1538 A/G) predicts the likelihood of renal disease progression from normo- to macroalbuminuria subjects. Mir et al. [32], examined the specific SNP (+1538 A/G) of *HSP-70* in patients from the similar population who were admitted for care of diabetic foot ulcers and reported that AG genotype of *HSP70-2* (OR 2.02; 95% CI: 1.02–4.01) was significantly associated with the severity of foot ulceration. To the best of our knowledge, this is the first report to demonstrate the association of *HSP70-2* (+1538 A/G) SNP with DN subjects. However, a similar study from the Polish population by Buraczynska et al. [33], reported on the association of *HSP70-2* (+1267 A/G) SNP with DN subjects, in which the authors had shown that the GG genotype and ‘G’ allele were significantly associated with DN (but not in T2DM), with an OR for the G allele of 4.77 (95% CI: 3.81–5.96). The disparity between the present study and the findings of Buraczynska et al. [33] is the variation in the polymorphic site analysed in *HSP70-2* gene. Moreover, the present study has explored the association of SNPs in different stages of nephropathy among South Indian population. In addition to the present findings on *HSP70-2* (+1538 A/G) SNP, our results showed that the GG genotypes of normoalbuminuria and macroalbuminuria subjects were found to have higher levels of total cholesterol and LDL-c than AA homozygotes. Thus, we suggest that this observed effect of the SNP might be associated with an increased risk of CVDs. These similar findings have previously been reported by Giacconi et al. [31], has the total cholesterol and LDL-c concentrations were significantly higher in B+ (presence of a G allele) than in B− patients with T2DM and atherosclerosis. However, this was not the objective of the present study and further investigation on the effect of these tested SNPs in subjects with and without CVD is required. Buraczynska et al. [33], also reported that the GG homozygotes of the *HSP70-2* (+1267 A/G) SNP had higher total cholesterol and LDL-c levels than AA homozygotes and suggested that this observed effect of the SNP might be associated with an increased risk of CVDs.

The *HSP70-hom* polymorphic (NcoI) site at nt 2437 corresponds to a methionine to threonine amino acid substitution at position 493 [17]. In the present study, the ‘T’ allele of *HSP70-hom* (+2437 C/T) SNP conferred significant protection for macroalbuminuria subjects. Our results are found to be inconsistent with an earlier report by Vargas-Alarcon et al. [19], who reported a significant association of the *HSP70-hom* +2437 ‘T’ allele with spondyloarthropathies in Mexicans. Another study by Martin et al. [34], reported that the presence of the HLA-B*5701 and *HSP70-hom* 493T alleles are necessary for the development of abacavir hypersensitivity. The *HSP70-hom* (+2437 T/C) SNP was investigated along with *HSP70-1* and *HSP70-2 gene* SNPs, but was found not to be involved in the susceptibility to Parkinson’s disease [18]. Buraczynska et al. [33] observed that no statistical significant differences in the genotypic distribution among patients with DN. These differences among different populations could be due to ethnic differences which have been well documented by genetic studies.

The present study on the effect of SNP and protein functionality analysis by computational prediction has shown that *HSP70-hom* (+2437 C/T) SNP was found to have only tolerated mutation. Even though the computational tool has predicted this mutation to be toleratable, the earlier report has shown that this particular SNP results in a Met  →  Thr amino acid substitution at position 493 in the peptide-binding domain and may affect the substrate specificity and chaperone activity of the HSP70-1L protein [35]. Hence, the study on protein level will help in determining the effect of this SNP on protein function.

## Conclusion

In summary, we analysed *HSP70-2* and *HSP70-hom* polymorphism in a highly homogeneous population with respect to ethnicity, geographic region and albuminuria subtypes. The present study showed that the ‘G’ allele of *HSP70-2* (+1538 A/G) SNP is associated with a significant risk of predicting a predisposition to macroalbuminuria in patients with T2DM, at least among South Indian population, while ‘T’ allele of *HSP70-hom* (+2437 C/T) SNP conferred significant protection against macroalbuminuria subjects. The present study could be replicated in different ethnic populations to gain more evidence. The strength of the present study is that all patients and controls are of the same ethnic origin. However, the major limitation of our study is its cross-sectional nature, which means that no cause-and-effect relationship can be drawn from the present study. Also, the present study did not examine the gene expression or the protein levels of HSP-70. Future studies aimed at describing this functional aspect will certainly aid our understanding of the role of these genes and their association with DN.

## Abbreviations

BMI: body mass index
CI: confidence interval
CVD: cardiovascular disease
DN: diabetic nephropathy
FPG: fasting plasma glucose
GWAS: genome-wide association studies
HDL-c: HDL cholesterol
HSP: heat-shock protein
KDM: Known Diabetes Mellitus
LDL-c: LDL cholesterol
NGT: Normal glucose tolerance
OHA: oral hypoglycaemic agent
OR: odds ratio
PPG: postprandial plasma glucose
SBP: systolic blood pressure
SNP: single nucleotide polymorphism
T2DM: type 2 diabetes mellitus
TGL: triglyceride
